# Hypoglycemia in patients with type 2 diabetes mellitus during hospitalization: associated factors and prognostic value

**DOI:** 10.1186/s13098-023-01212-9

**Published:** 2023-12-04

**Authors:** Tomás González-Vidal, Diego Rivas-Otero, Alba Gutiérrez-Hurtado, Carlos Alonso Felgueroso, Gema Martínez Tamés, Carmen Lambert, Elías Delgado-Álvarez, Edelmiro Menéndez Torre

**Affiliations:** 1 Department of Endocrinology and Nutrition, Hospital Universitario Central de Asturias/University of Oviedo, Oviedo, Spain; 2 Instituto de Investigación Sanitaria del Principado de Asturias (ISPA), Oviedo, Spain; 3 University of Barcelona, Barcelona, Spain; 4 Department of Medicine, University of Oviedo, Oviedo, Spain; 5 Centre for Biomedical Network Research on Rare Diseases (CIBERER), Instituto de Salud Carlos III, Madrid, Spain

**Keywords:** Type 2 diabetes, Hypoglycemia, Mortality, Prognosis

## Abstract

**Background:**

The risk factors for hypoglycemia during hospital admission and its consequences in patients with diabetes are not entirely known. The present study aimed to investigate the risk factors for hypoglycemia, as well as the potential implications of hypoglycemia in patients with type 2 diabetes mellitus admitted to the hospital.

**Methods:**

This retrospective cohort study included 324 patients (214 [66.0%] men; median age 70 years, range 34–95 years) with type 2 diabetes admitted to a university hospital who were consulted the Endocrinology Department for glycemic control during a 12-month period. We investigated the potential role of demographic factors, metabolic factors, therapy, and comorbidities on the development of in-hospital hypoglycemia. We explored the prognostic value of hypoglycemia on mortality (both in-hospital and in the long-term), hospital readmission in the following year, and metabolic control (HbA1c value) after discharge (median follow-up, 886 days; range 19–1255 days).

**Results:**

Hypoglycemia occurred in 154 (47.5%) patients during their hospitalization and was associated with advanced age, previous insulin therapy, higher Charlson Comorbidity Index, lower body mass index and lower baseline HbA1c values. Hypoglycemia was associated with greater in-hospital and long-term mortality, longer hospital stays, higher readmission rates, and poorer metabolic control after discharge. These negative consequences of hypoglycemia were more frequent in patients with severe (≤ 55 mg/dL) hypoglycemia and in patients who had hypoglycemia during a greater percentage of hospitalization days.

**Conclusions:**

Hypoglycemia during hospital admission is a marker of a poor prognosis in patients with type 2 diabetes.

**Supplementary Information:**

The online version contains supplementary material available at 10.1186/s13098-023-01212-9.

## Introduction

Hypoglycemia is defined as serum/plasma glucose values lower than 70 mg/dL, and its severity is classified according to the intensity of symptoms and blood glucose levels [1]. Hypoglycemia is a potential complication of type 2 diabetes mellitus (T2DM) therapy. Hypoglycemia is associated with higher morbidity and mortality [2]. Hence, preventing hypoglycemia is essential [3]. Several antidiabetic agents now available for the treatment of T2DM have low risk of hypoglycemia [4]. However, except in carefully selected cases, there is a recommendation to avoid the routine use of these drugs in hospitalized patients, maintaining insulin as the tool to control hyperglycemia in that setting [5]. Given that insulin is the drug with the greatest risk of hypoglycemia [4] and considering the clinical instability of patients admitted to the hospital, glycemic targets are less strict in hospitalized patients [5].

Previous studies have investigated risk factors for hypoglycemia during hospitalization in addition to insulin treatment [6–10], although few studies have been specifically focused on patients with T2DM [7, 9]. These studies showed a greater risk of hypoglycemia in patients with additional comorbidities and in patients who received other drugs with hypoglycemia risk, such as sulfonylureas [7–9]. However, these studies showed inconclusive results regarding the influence of glycemic control prior to admission on the appearance of hypoglycemia [6, 7]. Few studies have addressed the influence of antidiabetic drugs that are unlikely to produce hypoglycemia [11].

Although some studies have investigated the prognostic value of hypoglycemia in hospitalized patients [8, 9, 12–20], some have been performed on highly selected populations [8, 18–20]. In general, it is assumed that hypoglycemia predicts a longer hospital stay [9, 12–14, 16, 17, 20] and a higher in-hospital mortality [8, 12–20]. After adjusting for covariates, particularly comorbidities [8, 12, 14–19], the association between hypoglycemia and mortality has not always been maintained [8, 17]. Thus, it is not clear whether hypoglycemia is a risk factor for mortality by itself or whether it is a merely marker of the severity of the patient’s situation. In some studies, mortality was only related to spontaneous hypoglycemia and not to that caused by hypoglycemic treatment [18]. Several of these studies included patients with and without diabetes [8, 13, 15, 18, 19] and mixed patients with type 1 diabetes mellitus (T1DM) and T2DM [12, 14, 17]; two different diseases with different risk levels for hypoglycemia [21]. In studies that differentiated T1DM and T2DM [9, 16, 20], mortality in relation to hypoglycemia was lower in patients with T2DM than in those with T1DM [9, 16]. Few studies have investigated the potential relationship of hypoglycemia severity [12, 14, 15, 17] and frequency [14] with mortality. Furthermore, only a few studies have addressed the prognostic value of hypoglycemia during hospitalization for long-term mortality, with discordant results [8, 12, 14, 20]. Finally, to the best of our knowledge, no studies have been conducted to study the evolution of glycemic control at discharge of patients who had hypoglycemia during admission.

The present study aimed to investigate, specifically in patients with T2DM, the risk factors for development of hypoglycemia during hospitalization, as well as the potential influence on short- and long-term mortality and glycemic control of mild and severe hypoglycemia during the hospital stay.

## Materials and methods

### Study design and setting

This was a retrospective cohort study performed on patients hospitalized in the *Hospital Universitario Central de Asturias* (Spain) throughout the year 2020 for whom an assessment for glycemic control by the Endocrinology Department was requested during admission. Only patients with a diagnosis of T2DM were included, either known before admission or diagnosed during their hospital stay using American Diabetes Association (ADA) criteria [22]. We excluded patients without a prior diagnosis of T2DM who developed hyperglycemia due to corticosteroid treatment or after solid organ transplantation during hospitalization; pregnant patients; patients with T1DM (as evidenced by pancreatic islet autoantibodies or highly suspected due to debut in youth with low insulin reserve [22]); patients with diabetes secondary to pancreatic diseases; and patients in whom consultation was requested exclusively for glycemic control to perform 18F-fluoro-2-deoxy-D-glucose-positron emission tomography-computed tomography. A total of 324 patients (214 [66.0%] men; median age 70 years, range 34–95 years) met the criteria and were included in the study.

The standard hospital protocol for monitoring glycemic control in T2DM includes 3–8 capillary glycemia measurements per day, depending on the degree of control and the complexity of the treatment. The initial hypoglycemic treatment was chosen by the department that admitted the patient. The Endocrinology Department took over the treatment once it was consulted. Usual recommendations for treatment and therapy targets were followed [5], individualizing each case according to the patient characteristics.

### Main determinations

#### Outcomes

*In-hospital hypoglycemia.* A patient was considered to have had hypoglycemia when he/she had at least 1 capillary blood glucose measurement of less than or equal to 70 mg/dL during their stay. Similar to the ADA classification [22], mild hypoglycemia was defined as a blood glucose level between 56 and 70 mg/dL and severe hypoglycemia was defined as a blood glucose level less than or equal to 55 mg/dL. The total days of hospitalization with at least one determination in the hypoglycemia range were recorded, and based on these data, the percentage of hospitalization days with hypoglycemia was calculated using the total duration of hospitalization as the denominator.

*Length of hospital stay.* Measured in days, from the date of admission to the date of discharge.

*In-hospital and long-term mortality.* All-cause mortality during hospitalization was registered. For patients who did not die and were discharged, long-term mortality was recorded, with a follow-up extended until April 15, 2023. Survival in patients discharged alive from the hospital was calculated from the day of hospital discharge until death or until the last reliable contact with the healthcare system, to account for potential misclassification owed to change of residence. Median follow-up was 886 days (range 19–1255 days).

*Readmission.* For patients who did not die during hospitalization, readmission to any hospital service up to 1 year after discharge was recorded.

*Metabolic control after hospital discharge.* Baseline glycated hemoglobin (HbA1c) value was obtained during admission (or, failing that, the most recent previous value available). Follow-up HbA1c was the first available measurement after hospital discharge, provided that at a minimum of 2 months and a maximum of 12 months had elapsed after discharge.

#### Covariates

The following variables were collected from each patient: age; sex; serum glucose on admission; glomerular filtration rate on admission (calculated from serum creatinine levels using the CKD-EPI formula) [23]; body mass index (BMI); known T2DM diagnosis prior to admission according to the ADA criteria [22]; previous outpatient treatment with insulin; baseline HbA1c value (as described above); Charlson Comorbidity Index (CCI) as a global estimator of comorbidities [24]; hospital department/unit of admission (particularly, intensive care unit [ICU]); days of hospitalization elapsed until he/she was assessed by the Endocrinology Department; and treatment received during their stay (oral or intravenous corticosteroids, insulin, sulfonylureas, meglitinides, metformin, dipeptidyl peptidase-4 [DPP-4] inhibitors, glucagon-like peptide-1 [GLP-1] agonists, sodium-glucose cotransporter-2 [SGLT2] inhibitors, and thiazolidinediones).

### Statistical analyses

We employed the chi-squared test to compare proportions; the Mann–Whitney test to compare numerical data between independent groups; and the Wilcoxon test to compare paired samples of numerical values. We employed Spearman’s rank test to assess correlation, and used logistic regression for multivariate analysis of factors associated with hypoglycemia and with in-hospital mortality. A calculator and a nomogram with a visual scale method for prediction of in-hospital hypoglycemia using selected baseline variables was also constructed. The points to the variables were assigned in the calculator and in the nomogram scale based on the logistic regression model. We assessed the model’s diagnostic performance by constructing receiver operating characteristic (ROC) curves and evaluated them by calculating the area under the ROC curve. We employed Kaplan–Meier curves for an assessment of the mortality rates after hospital discharge according to hypoglycemia during admission and the log-rank test for between-group comparisons. Cox regression (proportional hazards regression) was used for multivariate analysis of survival during follow-up after hospital discharge. Covariates were forced to enter the equation in all multivariate models. All tests were two-tailed. P-values lower than 0.05 were considered statistically significant.

## Results

### Prevalence of hypoglycemia during hospital admission and associated risk factors

A total of 154 (47.5%) patients developed hypoglycemia during hospitalization; 63 (19.4%) presented only mild hypoglycemia, and 91 (28.1%) patients presented at least an episode of severe hypoglycemia.

Table 1 represents a comparison of patients with and without overall, mild and severe hypoglycemia during hospitalization. Advanced age, known previous diagnosis of diabetes, low HbA1c values, previous comorbidities (high CCI), insulin treatment during their stay, and previous outpatient insulin treatment were associated with both mild and severe hypoglycemia. Higher ambulatory doses of insulin, low BMI, decreased glomerular filtration rate, admission to a surgical unit, treatment with secretagogues during admission, and late evaluation by the Endocrinology Department were associated with severe hypoglycemia. ICU admission was associated with a higher frequency of hypoglycemia in general. Treatment with oral antidiabetics that are unlikely to cause hypoglycemia was associated with a lower frequency of hypoglycemia in general and a lower frequency of severe hypoglycemia, even when only patients who were treated with insulin (n = 302) were selected (P = 0.003 for hypoglycemia in general; P = 0.001 for severe hypoglycemia; see Additional file 1). There was no association between any form of hypoglycemia and sex, blood glucose on admission, or corticosteroid treatment during hospitalization (Table 1).

**Table 1:** Characteristics of patients with type 2 diabetes in relation to hypoglycemia during admission

Characteristic	No hypoglycemia (n = 170)	Mild hypoglycemia (blood glucose 56–70 mg/dL) (n = 63)	Severe hypoglycemia (blood glucose ≤ 55 mg/dL) (n = 91)	Any hypoglycemia (n = 154)
*Demographics*				
Age (years)	66 (57–75)	71 (65–77)*	74 (66–79)***	73 (66–78)***
Sex (male), n (%)	118 (69.4)	41 (65.0)	55 (60.4)	96 (62.3)
*Metabolic characteristics*				
Known diagnosis of type 2 diabetes before admission, n (%)	139 (81.7)	59 (93.6)*	88 (96.7)***	147 (95.4)***
Ambulatory treatment with insulin before admission, n (%)	56 (32.9)	43 (68.2)***	52 (57.1)***	95 (61.6)***
Insulin dose before admission (IU/kg/day)^a^	0.42 (0.23–0.63)	0.41 (0.27–0.61)	0.64 (0.44–0.91)**	0.57 (0.35–0.95)*
Multiple doses of insulin before admission, n (%)^b^	27 (50.0)	23 (53.4)	41 (78.8)**	64 (67.3)*
Body mass index (kg/m^2^)^c^	29.3 (25.6–34.1)	28.1 (24.0–32.5)	25.2 (22.4–29.1)***	25.8 (23.0–31.1)***
Serum glucose on admission (mg/dL)^d^	240 (182–349)	236 (142–311)	225 (160–309)	226 (152–310)
HbA1c during or before admission (%)^e^	8.1 (7.0–9.9)	7.6 (6.6–9.2)*	7.4 (6.5–8.9)**	7.5 (6.6–8.9)**
*Comorbidities*				
Charlson Comorbidity Index	5 (4–7)	6 (5–8)***	7 (6–9)***	7 (6–9)***
Estimated glomerular filtration rate (mL/min/1.7 m^2^)^f^	67 (40–87)	50 (31–84)	48 (27–76)**	48 (27–82)**
*Characteristics of admission*				
Surgical department admission, n (%)	48 (28.2)	26 (41.2)	38 (41.7)*	64 (41.5)*
ICU admission, n (%)	13 (7.6)	10 (15.8)	14 (15.3)	24 (15.5)*
Days until assessment by the Endocrinology department	5 (3–8)	7 (3–9)	8 (4–14)***	7 (4–13)***
*Therapy during admission*				
Insulin, n (%)^g^	150 (88.2)	61 (96.8)*	91 (100.0)***	152 (98.7)***
Secretagogues, n (%)^h^	13 (7.6)	6 (9.5)	16 (17.5)*	22 (14.2)
Antidiabetic agents that are unlikely to cause hypoglycemia, n (%)^i^	112 (65.8)	34 (53.9)	37 (40.6)***	71 (46.1)***
Systemic corticosteroids, n (%)^j^	75 (44.1)	31 (49.2)	49 (53.8)	80 (51.9)

Numerical variables are expressed as medians and interquartile ranges (within parentheses)

^*^P < 0.05; **P < 0.01; ***P < 0.001 versus patients with no hypoglycemia

^a^Data available for 110 patients (only patients with ambulatory treatment with insulin were included)

^b^Outpatient treatment with 2 or more insulin injections per day. Data available for 149 patients (only patients with ambulatory treatment with insulin were included)

^c^Calculated only if the body weight was available during admission. Data available for 282 patients

^d^Data available for 296 patients

^e^Preferably, HbA1c value during admission was used. If not available, the most recent HbA1c value available before admission was used. Data available for 320 patients

^f^Glomerular filtration rate was estimated by CKD-EPI equation, using the first creatinine value available during admission. Data available for 321 patients

^g^Patients who received at least one dose of any type of subcutaneous or intravenous insulin

^h^Includes sulfonylureas and meglitinides

^i^Includes metformin, DPP-4 inhibitors, GLP-1 agonists, SGLT2 inhibitors and thiazolidinediones

^j^Patients who received at least one dose of any type of oral or intravenous glucocorticoid

ICU, intensive care unit

Among baseline variables (Table 1), in-hospital hypoglycemia was independently and positively associated with older age (in years; odds ratio [OR] 1.04, 95% CI 1.01–1.06) and insulin therapy before admission (OR 3.04, 95% CI 1.77–5.21), and it was negatively associated with HbA1c (in percentage; OR 0.81, 95% CI 0.70–0.94) and BMI (in kg/m^2^; OR 0.93, 95% CI 0.89–0.97) on multivariate analyses (logistic regression). These 4 baseline variables allowed us to construct a nomogram and a calculator for probability prediction of hypoglycemia in patients with T2DM admitted to the hospital and assessed by an endocrinology department (see Additional files 2 and 3). The area under the ROC curve exploring the diagnostic performance of this model was 0.754 (95% CI 0.697–0.811).

### Longitudinal studies in relation to hypoglycemia during admission

#### Length of hospital stay

The patients who had hypoglycemia during hospitalization had longer hospital stays (mean, 27 days) than the patients who did not (median, 16 days; P < 0.001). There was a positive correlation between the days of hospitalization and the proportion of days of hospitalization in which there was at least 1 episode of hypoglycemia (Rho = 0.258; P < 0.001).

#### All-cause in-hospital mortality

A total of 30 (9.3%) patients died during hospitalization. Mortality was significantly higher in patients with hypoglycemia than in patients without it (Table 2). However, the mortality risk increase was almost restricted to the group of patients with severe hypoglycemia (Table 2). The patients who died during hospitalization had hypoglycemia during a higher percentage of days (mean, 9.7%) than the patients who did not (mean, 6.3%; P = 0.007). Among the 21 patients who did not receive insulin or secretagogues during their stay, only 2 developed mild hypoglycemia (which, therefore, could be considered spontaneous hypoglycemia). When patients who received insulin or secretagogues were selected (n = 303), the association between hypoglycemia (which could be considered as secondary to hypoglycemic treatment) and mortality during hospitalization was also maintained (P = 0.007; Additional file 1). The increased risk in hospital mortality in relation to hypoglycemia was still present after adjusting for age, sex, BMI, glomerular filtration rate, ICU admission, and CCI (Table 2).

**Table 2:** Risk of mortality in patients with type 2 diabetes in relation to hypoglycemia during admission

Level of hypoglycemia	No	Inpatient mortality (%)	Crude OR (95% CI)	Charlson Index- adjusted OR^a^ (95% CI)	Multi-adjusted OR^b^ (95% CI)
No hypoglycemia	170	4.7	1 (reference)	1 (reference)	1 (reference)
Any hypoglycemia	154	14.3	3.37 (1.45–7.82)**	2.90 (1.22–6.88)*	3.66 (1.33–10.0)*
Mild hypoglycemia (blood glucose 56–70 mg/dL)	63	4.8	1.01 (0.26–3.94)	0.89 (0.22–3.53)	1.30 (0.30–5.57)
Severe hypoglycemia (blood glucose ≤ 55 mg/dL)	91	20.9	5.34 (2.23–12.7)***	4.59 (1.87–11.2)***	6.03 (2.09–17.4)***

^*^P < 0.05; **P < 0.01; ***P < 0.001. OR, odds ratio

^a^Adjusted for Charlson Comorbidity Index [24]. Complete data available for 324 individuals

^b^Adjusted for age, sex, body mass index, glomerular filtration rate, and need for intensive care unit admission. Complete data were available for 279 individuals

#### All-cause long-term mortality

**Fig. 1:**
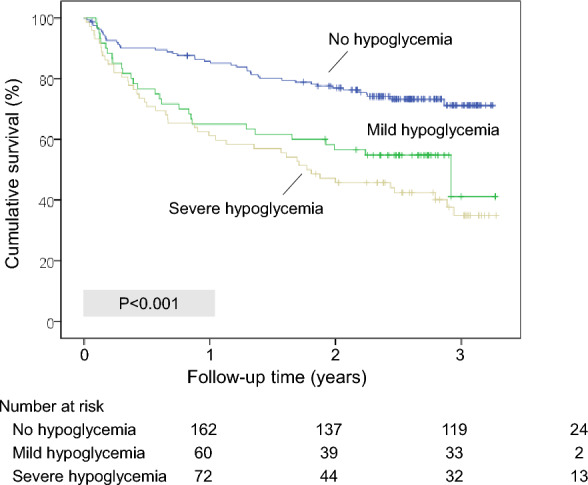
Probability of survival (Kaplan–Meier curves) in patients discharged from the hospital, stratified according to the presence or absence of hypoglycemia during admission (no hypoglycemia [overall mortality, 26.5%]; mild hypoglycemia [blood glucose 56–70 mg/dL, overall mortality, 46.6%]; severe hypoglycemia [blood glucose ≤ 55 mg/dL, overall mortality, 61.1%]). Vertical ticks represent censored data. P-value was calculated with the log-rank test

A total of 115 (39.1%) of 294 patients who were discharged alive from the hospital died during follow-up. Mortality during follow-up was higher among those who had developed hypoglycemia than in those who did not (Fig. 1). The patients who died during follow-up had had hypoglycemia during the previous admission for a greater percentage of days (mean, 9.4%) than the patients who did not (mean, 4.3%; P < 0.001). When patients who received insulin or secretagogues during their stay were selected (n = 273), the association between hypoglycemia (i.e., secondary to hypoglycemic treatment) and mortality after admission was still present (P < 0.001; Additional file 1).

On Cox regression analysis (proportional risk model), mortality risk among patients who had developed hypoglycemia during the previous admission was higher than that of the reference category (patients without hypoglycemia) (hazard ratio [HR] 2.48; 95% CI 1.70–3.63; P < 0.001). The association between in-hospital hypoglycemia and mortality risk was still present after adjusting for baseline CCI (HR 1.62; 95% CI 1.10–2.38; P = 0.013).

#### Metabolic control after hospital discharge

HbA1c follow-up was available from 177 patients discharged from the hospital. The level of HbA1c level tended to improve after hospital discharge in the majority of patients, although improvement was only significant among patients who did not develop hypoglycemia during their hospital stay: mean HbA1c decreased from 8.8% to 7.5% in patients without in-hospital hypoglycemia (n = 103; P < 0.001) and from 8.1% to 7.6% in patients with in-hospital hypoglycemia (n = 74; P = 0.204). In fact, HbA1c worsened in 26/103 (25.2%) patients without hypoglycemia during hospitalization and worsened in 35/74 (47.2%) with hypoglycemia during their stay (P = 0.002). Patients whose glycemic control (HbA1c level) at discharge worsened had had hypoglycemia for a greater percentage of days (mean, 8.6%) than patients whose glycemic control remained equal or improved (mean, 4.7%; P = 0.006). There was a positive correlation between the percentage of hospitalization days with hypoglycemia and the degree of change in HbA1c during the follow-up compared with baseline HbA1c (Rho = 0.219; P = 0.003).

#### Readmission after hospital discharge

Hospital readmission in the following year was more frequent among patients who had had hypoglycemia during the former hospital admission. Readmission occurred in 42/60 (70.0%) patients with mild hypoglycemia, 48/72 (66.7%) patients with severe hypoglycemia, and 75/162 (46.3%) patients without hypoglycemia during the former admission (P < 0.001). Patients who were readmitted during the following year had had a greater percentage of days with hypoglycemia during former hospitalization (mean, 7.7%) than patients who were not readmitted (mean, 4.5%; P < 0.001).

## Discussion

The present study shows that hypoglycemia in patients with T2DM, which is common during hospitalization (occurring in nearly a half of cases), is associated with increased in-hospital and long-term mortality, longer hospital stays, higher rates of readmission, and poorer metabolic control after discharge. To the best of our knowledge, this is the first T2DM-focused study to comprehensively demonstrate such associations. In addition, we have shown that these negative consequences of hypoglycemia are greater in patients with severe hypoglycemia and in patients who have had hypoglycemia during a greater percentage of hospitalization days, which had not been previously investigated.

The frequency of in-hospital hypoglycemia was significantly higher, as expected, than that observed in studies mixing patients with and without diabetes [8, 13, 15, 18, 19]. It should be considered that this is a selected sample of patients with T2DM who were complex enough for another specialist to consider an evaluation by an endocrinologist necessary. Similarly, this implies a greater frequency of glucose determinations and thus a greater probability that some of them fall into the hypoglycemic range.

The results regarding the factors associated with hypoglycemia in our series of patients with T2DM admitted to the hospital have similarities and differences with previous studies [6–10]. We confirmed that age, previous comorbidities (particularly, decreased glomerular filtration rate) and treatment during hospitalization with insulin or secretagogues are risk factors for hypoglycemia [6–10]. Hypoglycemia was less frequent in patients who received oral antidiabetics that are unlikely to cause hypoglycemia, even in those who were also treated with insulin. These patients were probably in a more stable clinical situation because, in general, clinical instability is a contraindication for their use [5]. This hypothesis is supported by the fact that patients who received these antidiabetic drugs had a lower CCI (P < 0.001; Additional file 1). Moreover, these oral antidiabetics could improve insulin resistance, thus implying a lower dose of insulin and therefore a lower risk of hypoglycemia. Low BMI was also associated with hypoglycemia in the present series, as assessed in previous studies [9, 10]. Low HbA1c values were also associated with hypoglycemia in our series. This contrasts with previous studies showing that hypoglycemia was more frequent in patients with HbA1c values greater than 9% [6]. Possibly, patients with already known diabetes, previous insulin therapy, and low baseline HbA1c values are prone to more intensive therapies during hospital admission, favoring hypoglycemia, although this hypothesis should be tested in future studies. In any case, our data allow the elaboration of a score based on 4 simple baseline variables (age, previous insulin therapy, BMI, and HbA1c value) that accurately predict the development of hypoglycemia in patients with T2DM admitted to the hospital who require consultation with an endocrinology department (Additional files 2 and 3). Early identification of patients who are at high risk for hypoglycemia can be useful in order to take steps to prevent hypoglycemia, such as the use of continuous glucose monitoring systems [25, 26].

We also observed that the frequency of hypoglycemia was higher the later that assessment by the Endocrinology Department occurred during admission. This relationship could be misleading, given that there was a directly proportional relationship between the days of admission and the days until the assessment by Endocrinology (Rho = 0.594; P < 0.001). Thus, it is possible that this higher frequency of hypoglycemia is explained by a longer average stay, and not necessarily because the intervention of a diabetes specialist significantly reduces the frequency of hypoglycemia. Likewise, the association between hypoglycemia and a longer hospital stay that had been reported in most studies [9, 12, 13, 16, 17, 20] could not indicate that hypoglycemia acts as a factor prolonging admission, as has been suggested, but that it could be the reverse, i.e., the more days the hospital stay lasts, the more blood glucose determinations will be performed and therefore more likely that some of them are compatible with hypoglycemia. However, our study suggests a direct relationship because it showed a positive correlation between the percentage of hospitalization days with at least 1 hypoglycemic event and the total admission days, as previously suggested [14].

Our study confirms the results of previous studies showing that hypoglycemia during hospitalization, insulin-mediated or not, is a marker of short-term mortality [8, 12–17, 19, 20]. Furthermore, it is one of the few studies that has analyzed the influence of hypoglycemia on long-term mortality [8, 12, 14, 20], showing that it remains a risk factor after a follow-up period of up to 3 years after hospital discharge. This association with mortality was still present after adjusting for covariates, particularly the CCI [24], an estimator of the patient baseline comorbidity and age. Regarding the mechanism of this association, it is difficult to argue that hypoglycemia during admission *per se* contributes to long-term mortality. Rather, it is likely that hypoglycemia is an additional marker of lability or disease severity leading to mortality. Future studies will be needed to elucidate the mechanisms by which hypoglycemia in hospitalized patients with T2DM is associated with short- and long-term mortality.

To the best of our knowledge, this is the first study to analyze the impact of hypoglycemia during hospitalization on glycemic control after discharge, documenting a worse glycemic control (in terms of HbA1c) in patients who had hypoglycemia while hospitalized. It could be argued that diabetes treatment intensity at discharge might have been reduced with the aim of preventing new hypoglycemia episodes. Furthermore, worsening of diabetes control could theoretically contribute to increased long-term mortality. However, the precise mechanisms for this finding deserve further studies.

The study has limitations that must be acknowledged, including those inherent to its retrospective design [27], particularly information biases and the possibility that confounders have not been adequately controlled. The distinction between adult T1DM and TD2M is not always straightforward, and adult patients with T1DM can be misclassified as T2DM [28]. Therefore, we cannot exclude the possibility that some patients with T1DM were misclassified as T2DM. It should also be considered that it is a selected population of patients who required consultation with an endocrinology department. As strengths, it should be noted that this is a study in real life, with prolonged follow-up, and performed exclusively in patients with T2DM, without mixing patients without diabetes or with T1DM.

## Conclusions

The present results could have practical clinical implications. The results might help predict the risk of hypoglycemia in patients with T2DM who are admitted to the hospital. Also, the results suggest that patients with T2DM who develop hypoglycemia when admitted to the hospital constitute a particularly vulnerable population, with a high in-hospital and post-discharge mortality, in which preventive measures should be emphasized. More studies will be needed to determine why hypoglycemia in patients with T2DM is associated with deleterious consequences in the short and long term.

### Supplementary Information

**Additional file 1.** A calculator for probability prediction of hypoglycemia in patients with type 2 diabetes admitted to the hospital and assessed by an endocrinology department.

**Additional file 2.** Data not shown in the main text.

**Additional file 3.** A proposed nomogram for the prediction of hypoglycemia in patients with type 2 diabetes admitted to the hospital and assessed by an endocrinology department. Age is included in years. Insulin: therapy with insulin before admission. HbA1c is included in percentage. BMI is included in kg/m2. Instructions: for the variable insulin therapy, the value “0” corresponds to “absent” and the value “1” is equal to “present”. Locate the factors on the respective axis and draw a line straight up to the points axis. Add the points for each of the factors and locate the final sum on the total points axis. Draw a line straight down to find the patient's probability of developing hypoglycemia.

## Data Availability

The datasets used and/or analyzed during the current study are available from the corresponding author on reasonable request.

## Abbreviations

ADA: American Diabetes Association
BMI: Body mass index
CCI: Charlson Comorbidity Index
DPP-4: Dipeptidyl peptidase-4
GLP-1: Glucagon-like peptide-1
HbA1c: Glycated hemoglobin
ICU: Intensive Care Unit
ROC: Receiver operating characteristic
SGLT2: Sodium-glucose cotransporter-2
T1DM: Type 1 diabetes mellitus
T2DM: Type 2 diabetes mellitus
