# Supplementary material for: Hypoglycemia in patients with type 2 diabetes mellitus during hospitalization: associated factors and prognostic value
Source: Diabetol Metab Syndr. 2023 Dec 4;15:249. doi: 10.1186/s13098-023-01212-9 (PMC10694969; doi:10.1186/s13098-023-01212-9)
Supplement: Supplementary file 1 — Additional file 1. A calculator for probability prediction of hypoglycemia in patients with type 2 diabetes admitted to the hospital and assessed by an endocrinology department. [file 13098_2023_1212_MOESM1_ESM.docx]

***Treatment with oral antidiabetics that are unlikely to cause hypoglycemia was associated with a lower frequency of hypoglycemia in general and a lower frequency of severe hypoglycemia, even when only patients who were treated with insulin (n=302) were selected (P=0.003 for hypoglycemia in general; P=0.001 for severe hypoglycemia; data not shown).***

Frequency of hypoglycemia in subjects under insulin treatment plus oral antidiabetics that are unlikely to cause hypoglycemia: 70/165 (42.4%).

Frequency of hypoglycemia in subjects under insulin treatment without oral antidiabetics that are unlikely to cause hypoglycemia: 82/137 (59.8%).

Frequency of severe hypoglycemia in subjects under insulin treatment plus oral antidiabetics that are unlikely to cause hypoglycemia: 37/165 (22.4%).

Frequency of severe hypoglycemia in subjects under insulin treatment without oral antidiabetics that are unlikely to cause hypoglycemia: 54/137 (39.4%).

***When patients who received insulin or secretagogues were selected (n=303), the association between hypoglycemia (which could be considered as secondary to hypoglycemic treatment) and mortality during hospitalization was also maintained (P=0.007; data not shown).***

Frequency of hypoglycemia in subjects under insulin or secretagogue treatment who died during admission: 22/30 (73.3%).

Frequency of hypoglycemia in subjects under insulin or secretagogue treatment who did not die during admission: 130/273 (47.6%).

***When patients who received insulin or secretagogues during their stay were selected (n=273), the association between hypoglycemia (i.e., secondary to hypoglycemic treatment) and mortality after admission was still present (P<0.001; data not shown).***

Frequency of hypoglycemia in subjects under insulin or secretagogue treatment who did not die during admission but died after admission: 71/110 (64.5%).

Frequency of hypoglycemia in subjects under insulin or secretagogue treatment who did not die during admission and did not die after admission: 59/163 (36.1%).

***Patients who received these antidiabetic drugs had a lower Charlson Comorbidity Index (P<0.001;*** ***data not shown).***

Charlson Comorbidity Index average in subjects under oral antidiabetics that are unlikely to cause hypoglycemia (n=183): 5.68 points.

Charlson Comorbidity Index average in subjects who did not receive antidiabetics that are unlikely to cause hypoglycemia (n=141): 6.80 points.
