# Supplementary figures and images for: Hypoglycemia in patients with type 2 diabetes mellitus during hospitalization: associated factors and prognostic value
Source: Diabetol Metab Syndr. 2023 Dec 4;15:249. doi: 10.1186/s13098-023-01212-9 (PMC10694969; doi:10.1186/s13098-023-01212-9)

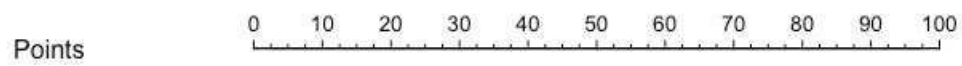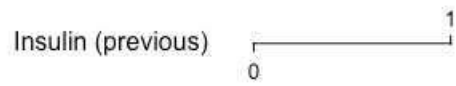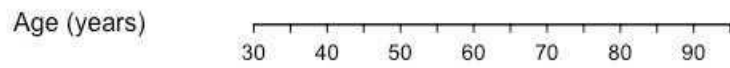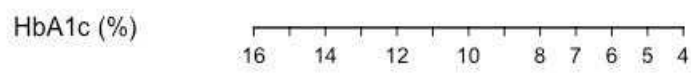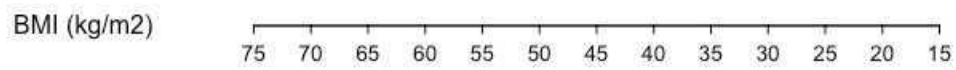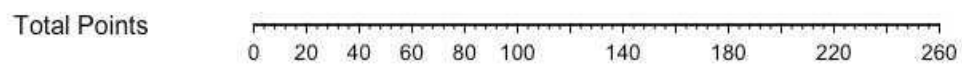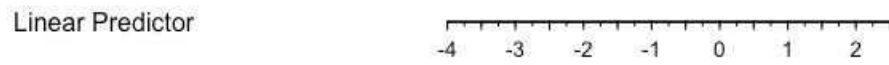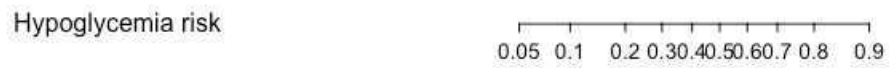

Supplement: Supplementary file 2 — Additional file 2. Data not shown in the main text. [file 13098_2023_1212_MOESM2_ESM.pdf]
